# Clinical outcomes of immune checkpoint inhibitor combined with other targeted or immunological therapy regimens for the treatment of advanced bile tract cancer: a systematic review and meta-analysis

**DOI:** 10.3389/fimmu.2024.1378760

**Published:** 2024-05-22

**Authors:** Jianpeng Zhou, Jia Li, Zhongqi Fan, Guoyue Lv, Guangyi Wang

**Affiliations:** ^1^ Department of Hepatobiliary and Pancreatic Surgery I, General Surgery Center, The First Hospital of Jilin University, Changchun, China; ^2^ Department of Hematology, The First Hospital of Jilin University, Changchun, China

**Keywords:** bile tract cancer, immune checkpoint inhibitor, combination therapy, treatment efficacy, adverse events

## Abstract

**Background and aims:**

A single immune checkpoint inhibitor (ICI) regimen has limited value in treating advanced bile tract cancer (BTC); therefore, ICI combination therapy is often applied. This meta-analysis aimed to evaluate the effectiveness and safety of ICI combination therapy for advanced BTC.

**Methods:**

The study protocol was registered on PROSPERO (CRD42023452422). Data on the median progression-free survival (PFS), median overall survival (OS), objective response rate (ORR), disease control rate (DCR), and grade ≥3 adverse events (AEs) reported in relevant studies were pooled and analyzed to determine the efficacy and safety of ICI combination therapy.

**Results:**

In total, 15 studies with 665 patients were included in this meta-analysis. The overall ORR and DCR were 34.6% and 77.6%, respectively. The overall median PFS and OS were 6.06 months [95% confidence interval (CI): 4.91–7.21] and 12.11 months (95% CI: 10.66–13.55), respectively. Patients receiving ICI combination therapy in addition to other therapies had a considerably prolonged median PFS and OS (z=9.69, *p*<0.001 and z=16.17, *p*<0.001). Patients treated as first-line treatment had a substantially longer median PFS and OS compared to patients treated as non-first-line treatment (z=11.19, *p*<0.001 and z=49.17, *p*<0.001). The overall pooled grade ≥3 AEs rate was 38.2% (95% CI: 0.268–0.497) and was not influenced by whether ICI therapy was combined with other treatments or not or the treatment line.

**Conclusion:**

Advanced BTC patients may benefit from ICI combination treatment without additional AEs. However, concurrent chemotherapy or radiotherapy is still needed to achieve better outcomes.

**Systematic review registration:**

https://www.crd.york.ac.uk/prospero/, identifier CRD42023452422.

## Introduction

Bile tract cancers (BTCs) represent a complicated category of epithelial malignancies and include gallbladder cancer (GC), intrahepatic cholangiocarcinoma (ICC), and extrahepatic cholangiocarcinoma (ECC). Despite being relatively rare, representing less than 1% of new cancer incidences worldwide (1), BTCs are becoming more prevalent (2). BTC has a poor prognosis with the 5-year overall survival (OS) rate estimated to be less than 20% (3), and when all stages of the disease are considered, treatment remains a challenge (4). Surgical resection is the only curative modality; however, only 20%–30% of BTCs are resectable at diagnosis (5). Given its sneaky onset and highly aggressive nature, early BTC diagnosis is elusive (6). Further, even for surgical candidates, though radical resection is still uncommon, the chance of relapse cannot be discounted (7).

In the past, chemotherapy was the primary treatment for advanced BTC. The ABC-02 research trial established the chemotherapy regimen of gemcitabine plus cisplatin (GC) as the first-line treatment for advanced BTC (8). Later, the ABC-06 trial demonstrated that a modified fluorouracil plus oxaliplatin (mFOLFOX) regimen may be a good option for second-line treatment after disease progression (9). However, the clinical outcomes for the above regimens were still dismal, with a median overall survival for first-line treatment of less than 12 months and about 6 months for second-line treatment.

Tumor immune checkpoint inhibitor (ICI) therapy has become a popular treatment option over the past ten years for various kinds of malignancies (10). By inhibiting immunological checkpoint molecules, such as programmed cell death protein 1 (PD1), programmed cell death ligand 1 (PDL1), and cytotoxic T lymphocyte antigen 4 (CTLA-4), ICI enables T-cell-mediated tumor cell killing and the elimination of regulatory T cells (Treg) (11, 12). Increased expression levels of PD1 and PDL1 have been detected in BTC tissue compared to non-tumor tissue, suggesting the use of anti-PD1/PDL1 might be effective in BTC (13). However, the objective response rate (ORR) to anti-PD-1 monotherapy was shown to be limited in early-phase studies (14, 15), ranging from 5.8%–22%. Consequently, ICI combination treatment has taken over as the most popular method of treating malignancies, usually as beyond first-line treatment regimens. For example, ICIs plus targeted agents, including antiangiogenic agents and multitargeted tyrosine kinase inhibitors (TKIs), or another ICI, have been reported in many single-arm studies for treating BTC (16, 17), and encouraging results have been reported. However, a meta-analysis of ICI combination treatment remains scarce.

Consequently, we carried out this meta-analysis to ascertain the treatment effectiveness and safety of combination ICI therapy for advanced BTC.

## Materials and methods

### Search strategy

The study protocol was registered on PROSPERO (CRD42023452422). This meta-analysis was carried out in accordance with the Preferred Reporting Items for Systematic Reviews and Meta-Analyses (PRISMA) criteria. A systematic search of the PubMed, Cochrane Library, Web of Science, Google Scholar, and Embase databases was performed to identify and retrieve literature in English that had been published from the time the databases were created until July 17, 2023. The following terms were used to search the databases to identify relevant literature: “biliary tract cancers”, “bile duct neoplasm”, “bile duct carcinoma”, “gallbladder carcinoma”, “cholangiocarcinomas”, “PD1”, “PDL1”, and “immunotherapy”. The American Society of Clinical Oncology (ASCO) and the European Society of Medicine Oncology (ESMO) reference lists for reviews and conference materials, such as abstracts and posters, up to June 31, 2023, were also manually searched.

### Selection criteria

The following were the study inclusion requirements: (1) clinical studies with human patients included; (2) patients pathologically diagnosed with BTC were treated with anti-PD1/PDL1 together with immunotherapy or other anti-PD1/PDL1, either combined with chemotherapy/radiotherapy or not; (3) progression-free survival (PFS), overall survival (OS), ORR, and adverse events (AEs) were reported; (4) quality studies based on the Newcastle–Ottawa Scale (NOS), with studies having a medium or high quality rating.

The following were the exclusion requirements: (1) editorials, letters, reviews, meta-analyses, protocols, and case reports; (2) either no comprehensive findings were supplied or the results were not clear; and (3) duplicate research. After identifying the initial papers, a full-text check was conducted to examine whether the papers met the inclusion and exclusion criteria. Two independent researchers performed the above processes, and their search results were consistent.

### Quality assessment

We used the NOS as a quality assessment indicator since most of the relevant research involved retrospective or single-arm studies. Studies with an NOS rating of 7–9 were considered high quality, while those with an NOS rating of 4–6 were considered medium quality.

### Data extraction

The target data were taken from the retrieved publications that were included in the study by two independent researchers. If disagreements existed, the other authors of this study collaborated to find solutions and reach agreement. The following information was taken out of the studies: names of the authors, year of publication, country, patient ages, total case numbers, the number of patients with ICC, ECC, and GC, the treatment regimens, the treatment line, and the median PFS, OS, and incidence of grade 3–5 AEs. If the PFS or OS was not described in detail (for example, the range of the OS was missing), the data were taken from Kaplan–Meier (K–M) curves using Engauge Digitizer V11.4.

### Statistical analysis

The PFS, OS, and treatment-related toxicity (AEs) were the primary endpoints. The above endpoint proportions were pooled and analyzed. The I^2^ value was used to assess the heterogeneity between studies. A random effect result was used with an otherwise fixed-effect outcome. I^2^ > 50% was deemed significantly heterogeneous. Sensitivity analysis was performed to find potential studies that could cause significant heterogeneity. A visual examination by funnel plot and quantitative analysis utilizing Egger’s test of the intercept were used to evaluate publication bias. All the statistical analyses were conducted with Stata (Version 14). A *p*-value of 0.05 was considered statistically significant.

## Results

### Search results and publication bias

After searching the aforementioned databases, 233 studies were initially identified. Among these, 28 studies were duplication studies, 10 studies were not clinical studies, 123 studies were deemed irrelevant, 52 studies had a sample size of <10, and 5 studies had no clinical outcomes, and these were excluded from our meta-analysis. Finally, 15 studies remained with 665 patients and these were included in our meta-analysis (16–30). **Supplementary Figure 1** shows the inclusion process flowchart for the studies.

### Quality assessment

Among the 15 included studies, there were 5 prospective studies (no randomized controlled trial) (16–18, 24, 25) and 10 retrospective studies (19–23, 26–30). All the studies underwent quality appraisal according to the NOS (**Supplementary Table 1**), with 13 ranked as medium quality, and 2 as high quality. Finally, the 15 studies all passed the quality inclusion criterion and were thus all included in the meta-analysis.

### Patient characteristics

The 15 included studies involved 665 patients in total, among whom 660 were finally included in the efficacy analysis in this meta-analysis, while the other 4 patients were excluded from the PFS and OS analysis but included in the adverse events analysis. The overall patient characteristics are presented in **Table 1**. Only 12 of the studies reported the patients’ median age, for which the pooled median age was 59.7 years old (range 25–82). Among the 661 patients, 434 (65.7%) patients had ICC, 43 (6.5%) ECC, and 184 (27.8%) GC. Besides ICI combination therapies, 177 (26.8%) patients received concurrent chemotherapy, 51 (7.7%) concurrent radiotherapy, and 23 (3.4%) locoregional therapy, which were not described in detail. In total, 251 patients (38.0%) received other treatments besides ICI. Moreover, 367 (55.5%) patients received ICI combination therapies as first-line treatment, and 298 (44.5%) received the therapies as non-first-line treatments.

**Table 1:** Characteristics of the included studies.

Author	Publication year	Study design	Country	Median age (range), y	Total patients (n)	Tumor location Intrahepatic/extrahepatic/gallbladder, n	Tumor size (mm), median, range	Regimes	Other treatment modality	Line of therapy	Clinical setting
Shi et al. (18)	2023	R	China	56.5 (25–73)	30	30/0/0	NR	Toripalimab combined with lenvatinib	Chemotherapy	First	Toripalimab (240 mg) intravenously every 2 weeks and oral lenvatinib (8 mg) once daily
Lei et al. (19)	2023	R	China	58.0 (50.5–69.0) and 60.0 (54.0–68.0)*	126	126/0/0	56.0 (36.5–80.0) and 60.0 (46.5–80.5)*	PD-1 inhibitors combined with TKIs	79 patients with chemotherapy	First	PD-1 inhibitors were administered intravenously every 3 weeks and TKIs orally daily
Wang et al. (20)	2023	R	China	61.0 (54.5–65.8)	31	19/4/8	NR	PD-1 inhibitors combined with lenvatinib	Radiotherapy	Second or beyond	Lenvatinib 8/12 mg orally at once daily. PD-1 inhibitor 200 mg (or 240 mg of toripalimab) every 3 weeks
Klein et al. (17)	2020	P	Australia	65 (37–81)	39	16/10/13	NR	Nivolumab combined with Ipilimumab	No	Second or beyond	Nivolumab 3 mg/kg and ipilimumab 1 mg/kg every 3 weeks for 4 cycles followed by nivolumab every 2 weeks
Shi et al. (21)	2022	R	China	62.5 (43–78)	74	35/4/35	NR	PD-1 inhibitors combined with lenvatinib	No	Second or beyond	Lenvatinib orally 12 mg/day daily. PD-1 intravenously administered (200 mg of sintilimab or tislelizumab or 240 mg of nivolumab or toripalimab) in a 3-week cycle
Wang et al. (22)	2023	P	China	<65 (26) >65 (14)	40	30/0/10	NR	Toripalimab plus lenvatinib	20 patients with radiotherapy	First	Lenvatinib orally 12 mg/day daily. PD-1 intravenously administered (200 mg of sintilimab or tislelizumab or 240 mg of nivolumab or toripalimab) in a 3-week cycle
Zhu et al. (23)	2023	R	China	59 (51–64)	57	30/9/18	NR	Lenvatinib combined with PD-1 inhibitors	Chemotherapy	25 with first and 32 with second or beyond	Lenvatinib orally 12 mg/day daily. PD-1 intravenously administered (200 mg of sintilimab or tislelizumab or 240 mg of nivolumab or toripalimab) in a 3-week cycle
Wang et al. (24)	2021	P	China	60 (39–72)	21	15/4/2	NR	Apatinib combined with camrelizumab	No	Second or beyond	Apatinib orally at 250 mg per day and camrelizumab intravenously 200 mg over every 3 weeks
Zhang et al. (25)	2021	P	China	62.50 (57.27–64.52)	38	20/5/13	NR	Lenvatinib combined with PD-1 inhibitors	No	First	Lenvatinib orally once daily and PD-1 inhibitor intravenously every 3 weeks
Ding et al. (26)	2022	R	China	59 (33–75)	41	41/0/0	86 (15–168)	Sintilimab combined with lenvatinib	23 patients with locoregional therapy	Second	Lenvatinib orally once daily and sintilimab 200 mg intravenously every 3 weeks
Xie et al. (27)	2022	R	China	53.0 (43.0–58.8)	40	40/0/0	6.7 (4.9–8.2)	Lenvatinib combined with PD-1 inhibitor	No	Second or beyond	Lenvatinib orally once daily and PD-1 inhibitor intravenously every 3 weeks
Zeng et al. (28)	2023	R	China	56.5 (33–69)	11	10/0/1	NR	PD-1/PD-L1 inhibitor combined with anlotinib	Chemotherapy	First	PD-1 inhibitor intravenously every 3 weeks and anlotinib (8–12 mg, day 1–14, orally, q3w)
Cousin et al. (16)	2022	P	France	63.1 (36–80)	29 (34)^#^	26/7/1	NR	Regorafenib combined with avelumab	No	14 with first and 20 with second or beyond	Avelumab intravenously every two weeks at a dose of 10 mg/kg. Regorafenib, 160 mg per day on a 3-week on/1-week off schedule
Wu et al. (30)	2023	R	China	69 (56–82)	52	0/0/52	NR	Anti-PD-1 inhibitor combined with lenvatinib	No	First	Lenvatinib 8 mg orally once daily and PD-1 inhibitor intravenously every 3 weeks
Zuo et al. (29)	2022	R	China	62 (58–69)	31	0/0/31	NR	Anti-PD-1 inhibitor combined with lenvatinib	No	First	Lenvatinib 8/12 mg orally once daily and PD-1 inhibitor intravenously every 3 weeks
Pooled				59.7 (25–82)	660	434/43/184			251 received other treatment	367 as first line	

P, prospective study; R, retrospective study; TKIs, tyrosine kinase inhibitors; BED, biological effect dose.

*The former data represented the patients who received ICI+targeted therapy and the latter data represented the patients who received ICI+targeted+chemotherapy.

#29 patients were finally included in the analysis.NR, Not reported.

### Efficacy

The main outcomes are presented in **Table 2**. All the studies, except that of Lei et al. (19), reported tumor responses (n = 535). Overall, only 3 patients (0.6%) achieved a complete response (CR), while 182 (34.0%) achieved a partial response (PR), and 230 patients (43.0%) had a stable disease (SD) state. The overall ORR and disease control rate (DCR) were 34.6% and 77.6%, respectively.

**Table 2:** Main outcomes extracted from the included studies.

Author	mFollow-up time (range), months	mPFS (95% CI), months	6m-PFS (95% CI), %	12m-PFS(95% CI), %	mOS (95% CI), months	6m-OS (95% CI), %	12m-OS (95% CI), %	ORR, % (95% CI)	DCR, %	CR, %	PR, n (%)	SD, %	Grade ≥3– AEs, %
Shi et al. (18)	23.5 (2.4–37.1)	10.2 (9.3–16.8)	NR	41.4	22.5 (15.6–29.3)	NR	76.7 (62.9–93.4)	80 (61.4–92.3)	93.3 (77.9v99.2)	1 (3.3)	23 (76.7)	4 (13.3)	50
Lei et al. (19)	14.6 (10.6–29.1)	7.2 (5.9–12.5) without chemotherapy and 6.9 (6.0–9.6) with chemotherapy	NR	NR	15.8 (11.7–24.8) without chemotherapy and 14.4 (12.3–NA) with chemotherapy	NR	63.7% (50.0%–81.1%) without chemotherapy and 63.8% (52.4%–77.8%) with chemotherapy	25.5% (13.9%–0.3%) without chemotherapy and 30.4% (20.5%–41.8%) with chemotherapy	NR	NR	NR	NR	10.6 without chemo therapy and 30.4 with chemotherapy
Wang et al. (20)	13.5	7.9 (7.1–8.7)	NR	NR	11.7 (8.3–12.9)	NR	NR	32.3 (14.8–49.7)	87.1 (74.6–99.6)	0	10 (32.3)	17 (54.8)	77.4
Klein et al. (17)	NR	2.9 (1.9–4.5)	NR	NR	5.7 (2.7–11.9)	NR	NR	23.1	43.6	0	9 (23.1)	8 (20.5)	15.4
Shi et al. (21)	15.0 (12.9–17.1)	4.0 (3.5–5.0)	18	NR	9.50 (9.0–11.0)	NR	23	71.62 (61.11–82.14)	20.27 (10.89–29.65)	0	15 (20.27)	53 (71.62)	52.7
Wang et al. (22)	NR	10.8 (6.2–15.4) with RT and 4.6 (3.3–5.8) without RT	NR	NR	13.7 (7.8–19.6) with RT and 9.2 (6.5–11.8) without RT	NR	NR	35 (12.1–57.9) with RT and 20 (0.8–39.2) without RT	85 (67.9–102.1) with RT and 75 (54.2–95.8) without RT	0	7 (35) with RT and 4 (20) without RT	10 (50) with RT and 11 (55) without RT	22 events with RT and 8 events without RT
Zhu et al. (23)	15.1 (IQR, 13.6–19.7)	9.27 (7.1–11.6)	71.8 (60.7–85.0)	29.6 (19.2–45.5)	13.4 (10.0–NA)	92.5 (85.6–99.8)	57.0 (44.5–73.1)	43.9 (31.8–56.7)	91.2 (81.1–96.2)	2 (3.5)	23 (40.4)	27 (47.4)	45.6
Wang et al. (24)	13.4 (11.9–14.8)	4.4 (2.4–6.3)	NR	NR	13.1 (8.1–18.2)	NR	NR	19.0 (7-40)	71.4 (50–86.1)	0	4 (19.0)	11 (52.3)	63.6
Zhang et al. (25)	13.7 (95% CI: 9.7–17.8)	8.0 (4.6–11.4)	63.2 (47.1–79.2)	21.1 (7.5–34.6)	17.7 (NR)	81.6 (68.7-94.5)	47.4 (30.7–64.0)	42.1 (25.7–58.6)	76.3 (62.2–90.5)	0	16 (42.1)	13 (34.2)	34.2
Ding et al. (26)	12.1 (5.1–19.1)	6.6 (4.9–8.3)	NR	NR	16.6 (5.0–28.2)	NR	NR	46.3 (30.7–62.6)	75.6 (59.7–87.6)	0	19 (46.3)	12 (29.3)	37.8
Xie et al. (27)	NR	5.83 (4.34–7.33)	32.5	NR	14.30 (11.76–16.84)	NR	61.4	17.5	75.0	0	8 (17.5)	23 (57.5)	17.5
Zeng et al. (28)	31.9 (23.8–39.7)	16.9 (7.0–NR)	90.9	54.5	16.9 (7.0–NR)	81.8	63.6	63.6	100	0	7 (63.6)	4 (36.4)	9.1
Cousin et al. (16)	9.8 (6.6–12.4)	2.5 (1.9–5.5)	27.6 (13.1–44.3)	6.9 (0.6–24.3)	11.9 (6.2–NA)	75.7 (53.5-88.3)	44.4 (19.0–67.3)	13.8	51.7	0	4 (13.8)	11 (37.9)	61.8
Wu et al. (30)	12.0 (IQR: 5.0–19.0)	7.0 (2.0–17.0)	NR	NR	12.0 (6.0–21.0)	NR	NR	46.2	65.4	0	24 (46.2)	10 (19.2)	9.6
Zuo et al. (29)	9.9 (IQR: 6.1–14.0)	5.0 (4.1–8.0)	44.9 (30–67.2)	NR	11.3 (9.6–12.3)	NR	48.7 (33.4–71)	32.3	83.9	0	10 (32.3)	16 (51.6)	67.7
Pooled													

mPFS, medium progression-free survival; CI, confidence interval; 6m-PFS, 6-month progression-free survival; 12m-PFS, 12-month progression-free survival; mOS, medium overall survival; 6m-OS, 6-month overall survival; 12m-OS, 12-month overall survival; ORR, objective response rate; DCR, disease control rate; CR, complete response; PR, partial response; SD, stable disease; AEs, adverse events; NR, not reported.

**Figure 1:**
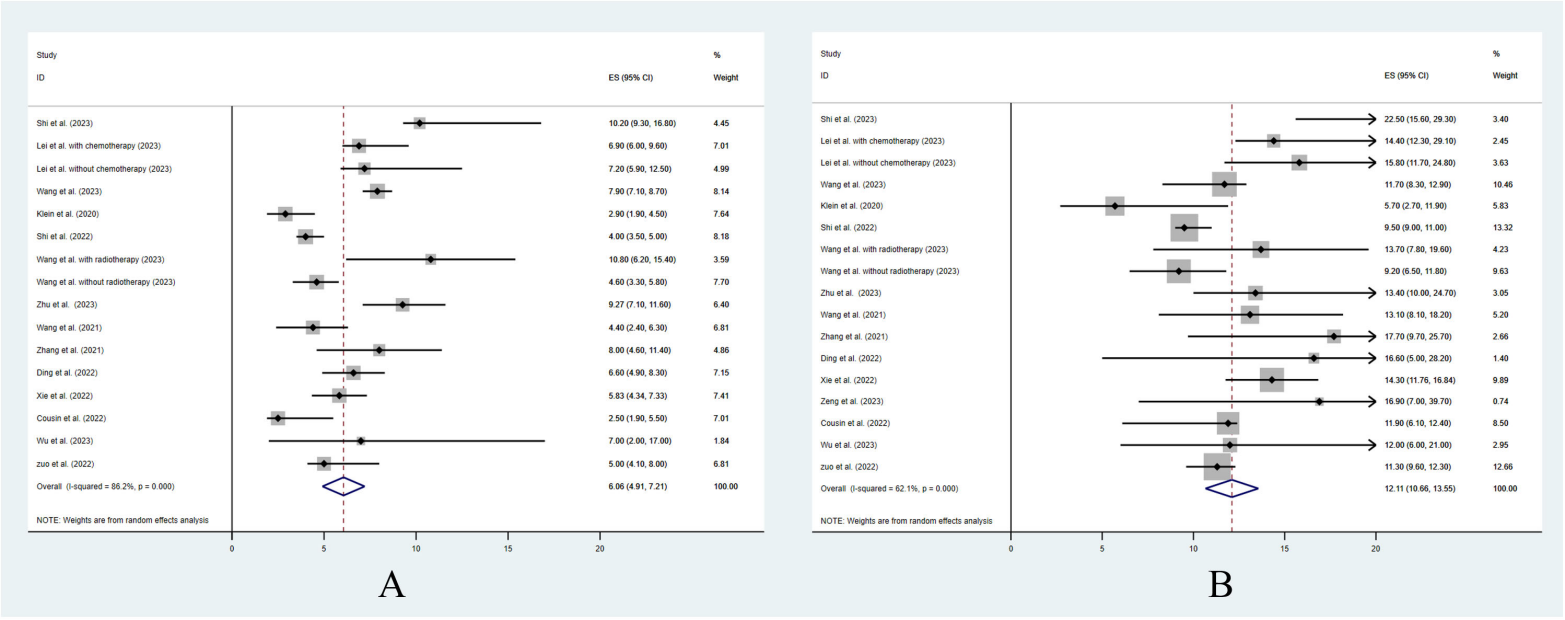
The pooled analysis of progression free survival **(A)** and overall survival **(B)**.

Twelve studies reported the median follow-up periods, which ranged from 9.8 to 31.9 months. The overall median PFS was 6.06 months [95% confidence interval (CI): 4.91–7.21] (**Figure 1A**). Only 7 studies reported the 6-month PFS, which was 49.6%. Also, only 5 studies reported the 12-month PFS, which was 20.1%. The median OS was 12.11 months (95% CI: 10.66–13.55) (**Figure 1B**). The 6-month OS was reported in 4 studies, and it was 87.7%. The 12-month OS was reported in 9 studies, and it was 51.5%.

**Figure 2:**
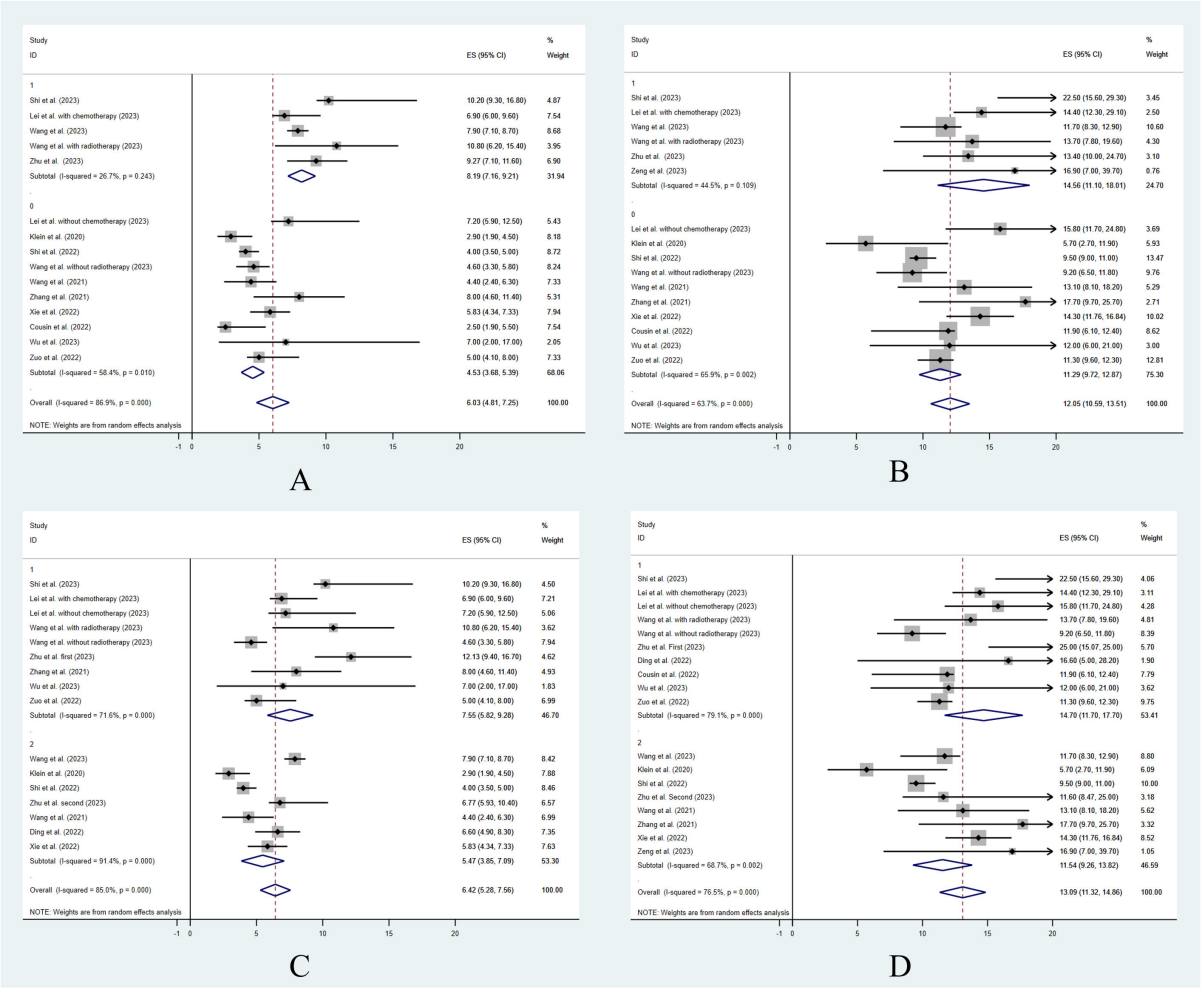
The treatment efficacy with different clinical situations. Progression free survival **(A)** and overall survival **(B)** for ICI combination treatment vs. other treatment. Progression free survival **(C)** and overall survival **(D)** for different treatment lines.

To analyze the effect of other treatments besides ICI combination therapy, 12 studies were included in the analysis. The study by Ding et al. (26) did not separately report the clinical outcomes of the patients who underwent other treatments or did not. Among the patients who were treated with ICI combination therapy alone, the estimated median PFS was 4.53 months (95% CI: 3.68–5.39), and the estimated median OS was 11.29 months (95% CI: 9.72–12.87). Among the patients who were treated with ICI combination therapy combined with other treatments, the estimated median PFS was 8.19 months (95% CI: 7.16–9.21) and the estimated median OS was 14.56 months (95% CI: 11.10–18.01). The median PFS and OS were significantly longer in patients treated with ICI combination therapy combined with other treatments (z = 9.69, *p* < 0.001, and z = 16.17, *p* < 0.001) (**Figures 2A, B**). The ORR and DCR for patients who were treated with ICI combination therapy combined with other treatments were 42.4% and 90.6%, respectively, while the ORR and DCR for patients who were treated with ICI alone were 28.7% and 71.3%, respectively. The ORR and DCR were thus significantly higher for patients who received other treatments as well (z = 18.3, *p* < 0.001, and z = 20.42, *p* < 0.001) (**Table 3**).

**Table 3:** Pooled results of ORR and DCR by treatment methods and treatment lines.

	ORR, n(%)	DCR, n(%)
Treatment methods		
ICI combination therapy alone (n=149)	73 (49.0)	135 (90.6)
ICI combination therapy combined with other treatments (n=345)	93 (27.0)	249 (72.2)
P-value	<0.001	<0.001
Treatment line		
First line (n=227)	108 (47.6)	184 (81.1)
Second line (n=278)	74 (26.6)	217 (78.1)
P-value	<0.001	0.407

To analyze the effect of the treatment lines, 12 studies were included in the statistical analysis that reported data on the treatment lines. The study by Cousin et al. (16) did not report the first-line and non-first-line clinical outcomes separately, and so was not included in the analysis. Among the patients who were treated as first line in the included studies, the estimated median PFS was 7.55 months (95% CI: 5.82–9.28) and the estimated median OS was 14.7 months (95% CI: 11.7–17.7). Among the patients who were treated as non-first line, the estimated median PFS was 5.47 months (95% CI: 3.85–7.09) and the estimated median OS was 11.54 months (95% CI: 9.26–13.82). The median PFS and OS were significantly longer in patients treated as first-line patients compared with non-first-line patients (z = 11.19, *p* < 0.001, and z = 14.17, *p* < 0.001) (**Figures 2C, D**). The ORR and DCR for patients who were treated as first-line patients were 47.6% and 81.1%, respectively. The ORR and DCR for patients who were treated as non-first-line patients were 26.6% and 78.1%, respectively. The ORR was significantly higher for patients who were treated as first line (z = 19.45, *p* < 0.001). However, the DCR showed no significant difference between the first-line and non-first-line patients (z = 1.42, *p* = 0.407) (**Table 3**).

### Safety

The study by Wang et al. (22) only reported the number of adverse events (AEs) that occurred, and it was not possible to ascertain the specific number of patients who experienced AEs. Therefore, the remaining 14 studies were included in the safety analysis.

**Figure 3:**
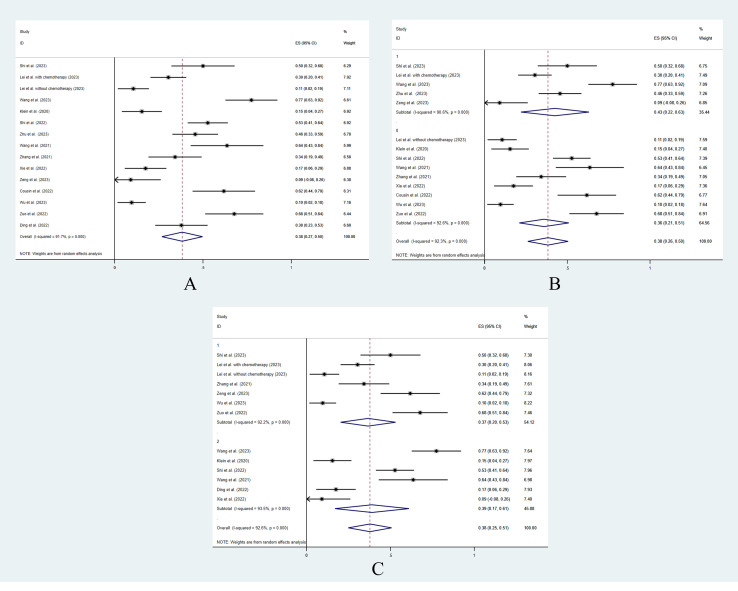
The pooled analysis of grade ≥3 adverse events. **(A)** Overall. **(B)** With or without additional concurrent treatments. **(C)** Different treatment lines.

The overall pooled grade ≥3 AEs rate was 38.2% (95% CI: 0.268–0.497) (**Figure 3A**). In patients who underwent concurrent chemotherapy or radiotherapy, the grade ≥3 AEs rate was 43.0% (95% CI: 0.22–0.63) and the grade ≥3 AEs rate in those who did not receive additional treatment was 36% (95% CI: 0.26–0.50). The grade ≥3 AEs rates showed no significant difference between the 2 groups (*p* = 0.10) (**Figure 3B**). Likewise, the treatment line did not influence the grade ≥3 AE rate (first line 36.7% vs. non-first line 39.0%, *p* = 0.24) (**Figure 3C**).

### Sensitivity analysis

Sensitivity analysis was performed leave one-out approach after the exclusion of studies with a high potential risk of publication bias from the meta-analyses. The stabilities of the PFS, OS, and AEs data were evaluated. According to the results of the sensitivity analysis conducted by STATE, 3 studies (16, 17, 28) were removed at a time to assess the PFS, and the median PFS was found to be 6.60 (95% CI: 5.44–7.75), and the results were comparable to the overall median PFS (6.60 *vs.* 6.06 months, *p* = 0.36).

Two studies (17, 18) were removed at a time to estimate the median OS, and the result was 11.84 months (95% CI: 10.61–13.08). The results were comparable to the overall median PFS (11.84 months vs. 12.11 months, *p* = 0.43).

Four studies (22, 28–30) were removed at a time to estimate the grade ≥3 AEs rates, and the result was 33.7% (95% CI: 0.22–0.43). The results were comparable to the overall grade ≥3 AEs rate (38.2% vs. 38.2%, *p* = 0.09).

The sensitivity analysis confirmed the stability of our findings.

### Publication bias

Egger’s tests were conducted for the median PFS, median OS, and grade ≥3 AEs rates (**Supplementary Figure 2**). The median PFS (*p* = 0.211) and median OS (*p* = 0.108) showed no publication bias. However, a possibility of publication bias (*p* = 0.016) was identified for the grade ≥3 AEs rates.

## Discussion

To the best of our knowledge, the present study is the first meta-analysis to evaluate the effectiveness and toxicity of ICI combination treatment in advanced BTC. We retrieved and analyzed data on the PFS, OS, tumor response, and safety of ICI combination treatment from the included study arms because the majority of trials in the literature studies were non-comparative. The pooled median PFS, OS, ORR, DCR, and grade ≥3 AEs of advanced BTC patients receiving combination treatment were 6.06 months, 12.11 months, 34.6%, 77.6%, and 38.2%, respectively. Moreover, we found in the subgroup analysis that the patients who received chemotherapy combined with/without radiotherapy had better treatment efficacy than those who did not. Likewise, the first-line treatment regimens had better treatment efficacy than the non-first-line treatment regimens. There were no discernible differences in the rates of severe AEs between the various arms.

For patients with unresectable BTC, the mainstay of treatment is still systemic chemotherapy., although the prognosis is still not satisfactory (8). ICIs have been licensed for the treatment of hepatocellular carcinoma, with ORRs varying from 15% to 20% (31). However, it has been reported that single immunotherapy drugs have limited action because of the unfavorable environment in BTC (32, 33). Also, in a number of randomized studies, anti-PD-1 monotherapy did not enhance the prognosis of BTCs that were unresectable (14, 15). A number of modest nonrandomized or single-arm studies with variations in the drugs used in ICI combination treatment have shown satisfactory or positive results (34–36). However, evidence on the effectiveness of ICI combined with other immunotherapy or targeted agents is still weak.

The tumor microenvironment (TME) in BTC is characterized by a dense stroma and a large population of different inflammatory and tumor cells (37). Numerous cells have been discovered to be associated with the immunoresistant microenvironment and desmoplastic reactions observed in BTC (37, 38). These cells have been linked to the spread of illness, immune evasion, and metastasis. One study showed that ICCs could be classified into four different TME-based molecular subtypes (39), with about half of the cases in that study immune desert subtypes, which are primarily resistant or tolerant to immunotherapy treatment (39). Two subtypes were proposed in an integrated genomic study by another group: the inflammation class and the proliferation class (40). According to that study, the inflamed/lymphoid subtype, which includes tumors with significant T-cell infiltration and immune checkpoint pathway activation, is related to the longest survival. This subtype ought to respond to immune checkpoint inhibitors more readily. The above results indicate that not all BTCs are sensitive to ICI and immunotherapy treatments.

Moreover, when compared to other solid tumors, BTCs seem to have a lower prevalence of tumor mutational burden-high (TMB-H), which may limit the efficacy of ICI treatment. In a study using next-generation sequencing on 164 Asian patients and 283 Western patients with ICC, the reported prevalence of TMB-H, defined as 10 mutations/Mb, was 12.2% and 5.9%, respectively (41). As a result, ICI therapy or immunotherapy alone may not be effective enough to improve the prognosis of BTC patients.

ICI combination therapies have also been studied in an effort to improve the therapeutic efficacy of single ICIs. Based on an assumed costimulatory effect of combination treatment, it is anticipated that anti-PD-1 or anti-PD-L1 and anti-CTLA-4 could help overcome the immune-resistant milieu and improve the prognosis (12). The mechanisms behind these combinations’ synergistic effects may be explained by a number of factors. First, the activation of an effective blockade can be enhanced by co-targeting T cells at the site of priming (12). In this situation, more cells will either receive stronger costimulatory signals or signals for cell activation. Second, the priming site has many T-cell targets, which may have an increased effect due to cell-extrinsic mechanisms (such as CD4 cells supporting CD8 effector cells). Third, although co-targeted, T cells have different spatiotemporal dynamics, which will contribute to the costimulatory signaling lasting longer (12). All of these mechanisms may contribute to the superior effectiveness of ICI combination therapies over single therapies. Therefore, we aimed to explore the clinical outcomes of ICI combination therapy in this study.

The results of our study were also promising when compared with using ICI as a single therapy. In the KEYNOTE-028 trial, pembrolizumab 10 mg/kg every two weeks was used to treat 23 advanced BTC patients (14). The ORR was 13%, and the median (95% CI) OS and PFS were 5.7 (3.1–9.8) and 1.8 (1.4–3.1) months, respectively, while the grade ≥3 AEs rate was 16.7%. In the KEYNOTE-158 trial, pembrolizumab 200 mg every three weeks was used. The ORR was 5.8% (6/104; 95% CI: 2.1%–12.1%), and the median (95% CI) OS and PFS were 7.4 (5.5–9.6) and 2.0 (1.9–2.1) months, respectively. Further, the ORR was 6.6% in individuals with tumors expressing a PD-L1 combination positive score ≥ 1 (14). Patients with a positive PD-1 expression level were enrolled in a different small prospective single-center cohort of 40 patients who received pembrolizumab as a second-line treatment (42). The median PFS and OS were 1.5 months (95% CI: 0.0–3.0) and 4.3 months (95% CI: 3.5–5.1), respectively, with an ORR of 10%. The results indicated the advantage of ICI combination therapy over ICI single therapy in advanced BTC.

Undoubtedly, there are still some limitations in our own study to note. First, the heterogeneity between studies caused by methodological and clinical diversities was high. Second, most of the studies were not comparative. Although the results are promising, the advantage of combination therapy should be verified in a further prospective randomized controlled study. Third, the effects of different regimens were not analyzed. Although their mechanisms may be the same, the effects of these regimens may be different. Fourth, the influence of the tumor location was not analyzed. Various anatomical subgroups have various approaches to treatment, biology, and epidemiology (6). However, most studies did not report clinical outcomes based on anatomical subtypes. Fifth, publication bias still existed in our study. This publishing bias was mostly caused by the overwhelming preferences of sponsors, publications, and researchers for the best results. Sixth, most of studies were conducted in China, which may have introduced bias in the study. A wider evaluation including more international studies and diverse populations would be warranted to address this bias. In the future, large-scale prospective studies with a strict design are needed to address the heterogeneity among different studies and further investigate the use of ICI combination therapy in treating BTC. We hope our work will inspire and prompt further work in this area.

## Conclusion

After PFS, OS, ORR, DCR, and severe AEs rates from the studies were pooled and analyzed, our meta-analysis demonstrated that ICI combination therapy can be an efficacious and safe therapeutic option. However, concurrent chemotherapy or radiotherapy is still needed to achieve better outcomes. Further prospective RCT studies comparing ICI combination therapy and other treatment regimens may be needed to establish the true role of ICI combination therapy in treating BTC, which will facilitate the development of combination drug regimens.

## Data availability statement

The original contributions presented in the study are included in the article/**Supplementary Material**. Further inquiries can be directed to the corresponding author.

## Author contributions

JZ: Investigation, Methodology, Writing – original draft. JL: Data curation, Project administration, Visualization, Writing – original draft. ZF: Data curation, Resources, Validation, Writing – original draft. GL: Conceptualization, Validation, Writing – review & editing. GW: Investigation, Methodology, Validation, Visualization, Writing – review & editing.
