# Supplementary material for: Clinical outcomes of immune checkpoint inhibitor combined with other targeted or immunological therapy regimens for the treatment of advanced bile tract cancer: a systematic review and meta-analysis
Source: Front Immunol. 2024 May 22;15:1378760. doi: 10.3389/fimmu.2024.1378760 (PMC11150610; doi:10.3389/fimmu.2024.1378760)
Supplement: Supplementary file 1 [file Table_1.docx]

**Supplementary Table 1** Quality evaluations of all the included articles according to the Newcastle–Ottawa scale

| **Author** | **Year** |  | **Selection** | | | |  | **Comparability** | |  | **Outcome** | | |  | **Total score** |
| --- | --- | --- | --- | --- | --- | --- | --- | --- | --- | --- | --- | --- | --- | --- | --- |
|  |  |  | **(1)** | **(2)** | **(3)** | **(4)** |  | **(1)** | **(2)** |  | **(1)** | **(2)** | **(3)** |  |  |
| Shi et al.(18) | 2023 |  | **🟑** | - | **🟑** | **🟑** |  | - | - |  | **🟑** | **🟑** | **🟑** |  | 6 |
| Lei et al. (19) | 2023 |  | **🟑** | **🟑** | **🟑** | **🟑** |  | **🟑** | **🟑** |  | - | **🟑** | **🟑** |  | 8 |
| Wang et al.(20) | 2023 |  | **🟑** | - | **🟑** | **🟑** |  | - | - |  | **🟑** | **🟑** | **🟑** |  | 6 |
| Klein et al.(30) | 2020 |  | **🟑** | - | **🟑** | **🟑** |  | - | - |  | **🟑** | **🟑** | **🟑** |  | 6 |
| Shi et al.(21) | 2022 |  | **🟑** | - | **🟑** | **🟑** |  | - | - |  | **🟑** | **🟑** | **🟑** |  | 6 |
| Wang et al.(22) | 2023 |  | **🟑** | - | **🟑** | **🟑** |  | - | - |  | **🟑** | **🟑** | **🟑** |  | 6 |
| Zhu et al. (23) | 2023 |  | **🟑** | - | **🟑** | **🟑** |  | - | - |  | **🟑** | **🟑** | **🟑** |  | 6 |
| Wang et al.(24) | 2021 |  | **🟑** | - | **🟑** | **🟑** |  | - | - |  | **🟑** | **🟑** | **🟑** |  | 6 |
| Zhang et al.(25) | 2021 |  | **🟑** | - | **🟑** | **🟑** |  | - | - |  | **🟑** | **🟑** | **🟑** |  | 6 |
| Ding et al.(26) | 2022 |  | **🟑** | - | **🟑** | **🟑** |  | - | - |  | **🟑** | **🟑** | **🟑** |  | 6 |
| Xie et al(27) | 2022 |  | **🟑** | - | **🟑** | **🟑** |  | - | - |  | **🟑** | **🟑** | **🟑** |  | 6 |
| Zeng et al(28) | 2023 |  | - | **🟑** | **🟑** | **🟑** |  | **🟑** | **🟑** |  | **🟑** | **🟑** | **🟑** |  | 8 |
| Cousin et al. (16) | 2022 |  | **🟑** | - | **🟑** | **🟑** |  | - | - |  | **🟑** | **🟑** | **🟑** |  | 6 |
| Wu et al.(30) | 2023 |  | **🟑** | - | **🟑** | **🟑** |  | - | - |  | **🟑** | **🟑** | **🟑** |  | 6 |
| Zuo et al.(29) | 2022 |  | **🟑** | - | **🟑** | **🟑** |  | - | - |  | **🟑** | **🟑** | **🟑** |  | 6 |

**Selection 0-4🟑**

(1) Representativeness of the exposed cohort.

(2) Selection of the non-exposed cohort.

(3) Ascertainment of exposure.

(4) Demonstration that the outcome of interest was not present at the start of the study.

**Comparability 0–2🟑**

Comparability of cohorts on the basis of the design or analysis

(1) Study controls for _____________ (select the most important factor)

(2) Study controls for any additional factor (This criteria could be modified to indicate a specific control for a second important factor.)

**Outcome 0–3🟑**

(1) Assessment of the outcome

(2) Was follow-up long enough for the outcomes to occur?

(3) Adequacy of follow up of the cohorts
