# Supplementary material for: Clinical outcomes of immune checkpoint inhibitor combined with other targeted or immunological therapy regimens for the treatment of advanced bile tract cancer: a systematic review and meta-analysis
Source: Front Immunol. 2024 May 22;15:1378760. doi: 10.3389/fimmu.2024.1378760 (PMC11150610; doi:10.3389/fimmu.2024.1378760)

**Supplementary Figure 1.** Flowchart of the study selection process.

**
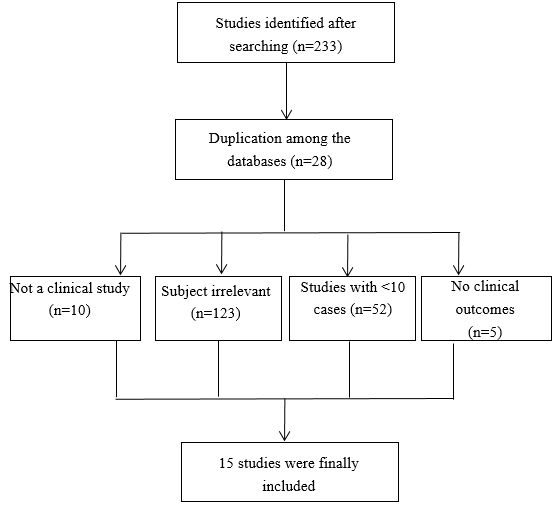
**

**Supplementary Figure 2.** The Egger’s test results for the median PFS(A), median OS(B), and grade ≥3 AE rates(C) to explore the publication bias of the study.


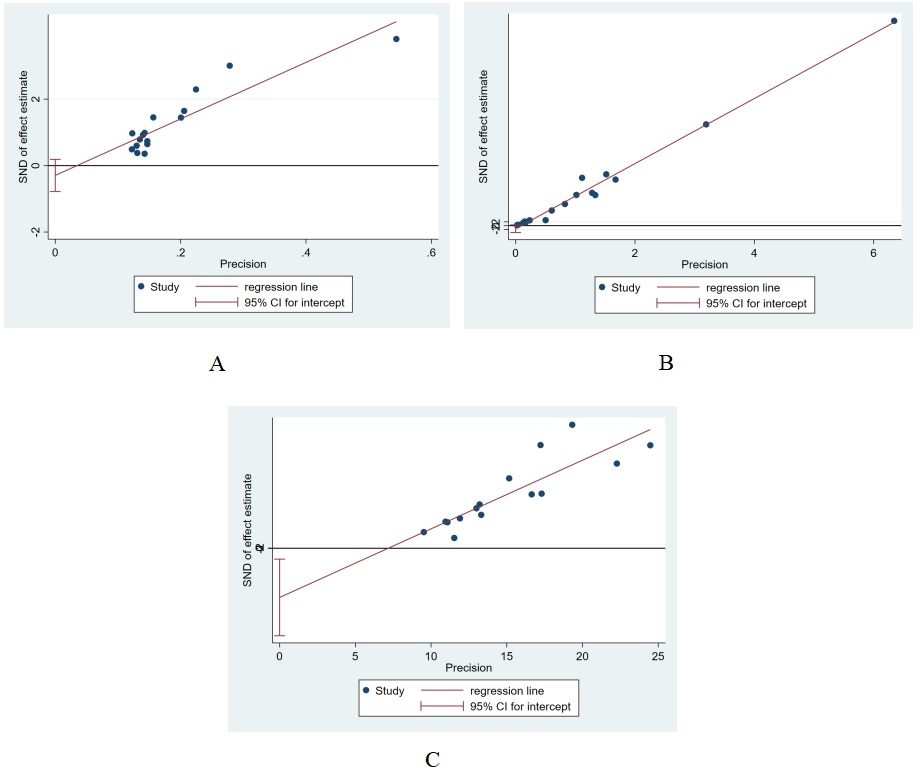

Supplement: Supplementary file 2 [file DataSheet_1.docx]
